# Comparative Evaluation of 0.9% Normal Saline Versus Acetate-Gluconate-Based Balanced Solution on Acid-Base Status and Postoperative Liver Function in Donor Hepatectomy Patients

**DOI:** 10.7759/cureus.69094

**Published:** 2024-09-10

**Authors:** Sonam Satija, Shashank Pandey, Neetu Jain, Jayashree Sood

**Affiliations:** 1 Department of Anesthesiology, Institute of Liver and Biliary Sciences, Delhi, IND; 2 Department of Anesthesiology, Pain and Perioperative Medicine, Sir Ganga Ram Hospital, New Delhi, IND

**Keywords:** acid-base balance, hepatectomy, intravenous fluid therapy, live donor liver transplantation, postoperative liver function

## Abstract

Background

Live donor liver transplantation, a widely practiced procedure, involves resecting a portion of a healthy donor’s liver for transplantation. Despite advancements, it poses challenges like cardiovascular instability and electrolyte imbalances, with maintaining acid-base balance being critical. This study compares the effects of 0.9% normal saline and PlasmaLyte A® on acid-base status and postoperative liver function.

Methodology

This prospective observational study was conducted over one year among 40 healthy adults aged 18-60 years undergoing donor hepatectomy. Patients were alternately allocated to receive either 0.9% saline (Group 1; n = 20) or PlasmaLyte A® (Group 2; n = 20). Key parameters, including acid-base status, hemodynamic parameters, and postoperative liver function, were monitored at various intervals. Statistical analysis was performed using IBM SPSS Statistics for Windows, Version 25.0 (Released 2017; IBM Corp., Armonk, NY, USA), with appropriate statistical tests. A p-value <0.05 was considered statistically significant.

Results

The study included 40 patients, with 20 in each group. No significant differences were observed between the groups concerning age, gender, weight, hemodynamic parameters, and urine output. However, significant differences were found in acid-base parameters. Group 2 showed better preservation of acid-base balance with higher pH and HCO₃ levels. Patients in Group 1 exhibited a significant decrease in HCO₃ levels during surgery, while those in Group 2 maintained a more stable metabolic profile. Furthermore, nine patients in Group 1 required sodium bicarbonate supplementation for metabolic acidosis, compared to only three in Group 2. Postoperative liver function tests revealed no significant differences between the two groups, although a trend toward better outcomes was observed in Group 2.

Conclusions

PlasmaLyte A® demonstrated superior preservation of acid-base balance compared to 0.9% normal saline, with less need for bicarbonate supplementation. While liver function outcomes were similar, the balanced solution showed a trend toward better metabolic stability, suggesting it may offer safer and more effective fluid management in liver transplantation surgery.

## Introduction

Liver transplantation remains a critical and life-saving intervention for patients with end-stage liver disease. The success of this procedure heavily depends on obtaining an optimal donor graft with adequate volume. Live donor liver transplantation is now widely practiced globally. Live donor hepatectomy involves the surgical resection of part of a healthy donor’s liver for transplantation into a patient with end-stage liver disease [1]. In 2021, a total of 34,944 liver transplants were performed globally, with 23% involving living donors. The highest liver transplant rates per million population were observed in Korea, the United States, and several Western European countries, as per the Global Observatory on Donation and Transplantation [2]. Advancements in perioperative management, surgical techniques, anesthetic expertise, and intraoperative monitoring have improved the outcomes of liver transplants [1]. However, perioperative management of patients undergoing donor hepatectomy is associated with challenging potential complications such as cardiovascular instability, blood loss, coagulopathy, and electrolyte imbalances. One of the frequently encountered issues in these patients is maintaining acid-base balance and decongesting the liver for easier resection. Underperfusion can lead to anaerobic metabolism and increased lactate levels. Additionally, the reduction of functional liver cell mass post-surgery may contribute to hyperlactemia [3]. Intravenous fluid therapy is of growing interest due to its implications on acid-base balance. The choice of intravenous fluid therapy is often arbitrary, with various crystalloid solutions differing in composition. Normal saline (0.9% NaCl solution) is the most frequently prescribed crystalloid in clinical practice. However, due to its nonphysiological ion content and lack of buffering capacity, normal saline infusion can result in metabolic acidosis. A study showed a significant association between acute kidney injury and hyperchloremic acidosis with an increase in morbidity [4]. These effects can be mitigated by using a solution more similar to plasma’s aqueous fraction, such as PlasmaLyte, which contains acetate and gluconate, which can avoid acidosis by including bicarbonate or metabolizable anions. Additionally, conventional coagulation markers often become deranged after donor hepatectomy, with postoperative coagulopathy typically peaking between days 2 and 5. This condition is generally self-limiting, resolving without the need for fresh frozen plasma transfusion in donors [5-8]. Since there is limited data on the comparative effects of 0.9% normal saline and acetate-gluconate-based solutions on acid-base status in donor hepatectomy [9], we aimed to evaluate their impact on acid-base status and postoperative liver functions so as to guide clinical practice toward a safer and more effective fluid management strategy in donor hepatectomy surgeries.

## Materials and methods

This prospective observational study was conducted in the Department of Anesthesiology, Pain and Perioperative Medicine at Sir Ganga Ram Hospital, New Delhi, India, over a period of one year, after approval from the Institutional Ethics Committee and Organ Transplant Committee, among all consecutive healthy adults aged 18-60 years undergoing living donor hepatectomy with the objective to assess the effect of two different types of intravenous fluid on acid-base balance and postoperative liver functions.

The sample size was based on finding a significant difference in acid-base balance in the two groups. From our hospital experience, the mean base excess of five patients was calculated as five in the normal saline group. With reference to a previous study by Hofmann-Kiefer et al. [10], 15 patients per group were selected, thus providing a 90% power for detecting a difference of 3.0 for base excess between the two groups at an alpha level of 0.05 where the standard deviation was 2.5.

The formula for calculating the sample size is as follows: (n = (σ_1_^2^ + σ_2_^2^). [Z 1- α/2 + Z 1- β]^2 ^/(M1 - M2)^2^ = (6.25 + 6.25). [1.96 + 1.282] ^2^/ (3*3) = 12.5*10.510/9 = 131.38/9 = 14.60), where Zα/2 is the critical value of the normal distribution at α/2 (e.g., for a confidence level of 95%, α is 0.05 and the critical value is 1.96), Zβ is the critical value of the normal distribution at β (e.g., for a power of 90%, β is 0.1 and its critical value is 1.282), and σ 1 and σ 2 are the standard deviations of the two groups. M1 and M2 are the means of two groups. We rounded it off to 20, i.e., 20 patients in each group, hence a total of 40 patients.

Inclusion criteria were ASA grade 1 and 2 patients aged 18-60 years undergoing elective donor hepatectomy. Exclusion criteria included patients with hypertension, cardiac disease, diabetes mellitus, underlying metabolic disorders, or those who did not consent.

Informed consent was obtained from patients, and participants were alternately allocated to receive either 0.9% saline (Group 1) or an acetate-gluconate-based solution (PlasmaLyte A®) (Group 2). Preanesthetic examinations included a detailed history and physical examination, with a focus on the cardiovascular and respiratory systems. The airway was evaluated by modified Mallampati grading, and routine and special investigations were ordered as indicated. Patients were kept fasting for eight hours prior to surgery and premedicated with alprazolam (0.25 mg) the night before and ranitidine (150 mg) on the morning of surgery.

Venous access was secured using a 20G cannula, and intravenous fluids were started. Standard ASA monitoring, including ECG, non-invasive blood pressure, heart rate, end-tidal CO2, and pulse oximetry, was initiated in the operating room. General anesthesia was induced with fentanyl citrate (2-3 µg/kg), propofol (1.5-2.5 mg/kg), and atracurium besylate (0.6 mg/kg). Patients were ventilated to maintain EtCO2 between 35 and 40 mm Hg, along with an epidural catheter placed at the T7/8 interspace and a central venous catheter inserted into the right internal jugular vein. An arterial catheter was placed in the radial artery for hemodynamic monitoring and blood sample collection.

Intravenous fluid (0.9% saline for Group 1, PlasmaLyte A® for Group 2) was started at 5 ml/kg/hr for maintenance, with the infusion rate adjusted to maintain a low central venous pressure of 2-4 mm Hg during liver resection and increased to 8-12 mm Hg post-resection. Packed RBCs were transfused to maintain a hematocrit above 25%. Arterial blood samples were analyzed for acid-base balance at baseline (T0), start of resection (T1), end of resection (T2), surgical closure (T3), immediate postoperative (T4), and 24 hours postoperatively (T5) using an EPOCÒ arterial blood gas analyzer. Hemodynamic parameters were recorded at the same time points.

Postoperatively, patients were extubated and transferred to the liver intensive care unit, where they received 5% dextrose at 100 ml/hr. Liver function tests, including serum bilirubin, SGOT, and prothrombin time, were conducted at D0, D1, D2, D3, D4, and D5.

Variations in all parameters were evaluated through both intergroup and intragroup comparisons. Statistical analysis was performed using IBM SPSS Statistics for Windows, Version 25.0 (Released 2017; IBM Corp., Armonk, NY, USA). Continuous variables were presented as mean ± SD and compared using the unpaired t-test, while categorical variables were analyzed using the chi-square test or Fisher’s exact test. Repeated measures were analyzed using repeated measures ANOVA with Bonferroni correction for paired comparisons, with a p-value of <0.05 considered statistically significant.

## Results

The study included 40 patients, with 20 patients in each group. The characteristics of the study population are depicted in Table 1. The gender distribution, mean age, and weight were comparable in both groups. As shown in Table 2, no significant difference was observed between the two groups with respect to the hemodynamic parameters and urine output. Acid-base parameters were also studied at various intervals, as depicted in Table 3. A statistically significant difference in pH (p = 0.000) was found at the end of 24 hours (T5) between the two groups. Although the mean pH remained within normal limits in both groups, it was higher in Group 2. There was also a significant difference in HCO₃ levels between the two groups at T2 (p = 0.036), T3 (p = 0.000), T4 (p = 0.000), and T5 (p = 0.024). HCO₃ remained low in group 1 throughout the surgery. The lowest mean HCO₃ was observed in Group 1 during T3, and the highest mean HCO₃ was observed in Group 2 at T5. There was a significant decrease in HCO₃ levels in the normal saline group at T2, T3, and T4 time points as compared to baseline (T0). A decreasing trend of anion gap was noted in Group 1, which was statistically significant at T4 (p = 0.034). Both groups were comparable at baseline with regard to base excess. However, base excess decreased in Group 1, which was statistically significant at T2 (p = 0.005), T3 (p = 0.001), T4 (p = 0.041), and T5 (p = 0.027) as compared to Group 2. Base excess was relatively better preserved in Group 2. The serum electrolyte levels at various intervals have been depicted in Table 4. Both groups had comparable sodium levels in baseline ABG post-induction. Sodium showed an increasing trend in group 1 at T2 (p = 0.004), T3 (p = 0.002), and T4 (p = 0.011), which was statistically significant. The mean highest sodium noted in Group 1 was 138.43 mmol/L at the T3 interval. Group 1 showed an increasing trend of chloride levels, and the difference in chloride in the two groups was statistically significant at T1 (0.000), T2 (0.000), T3 (0.000), T4 (0.000), and T5 (0.000). Group 1 showed a significant intragroup rise in chloride levels over time in comparison to baseline T0. The highest mean chloride concentration was noted in Group 1 at the T3 interval. The values remained within the normal range in Group 2. The two groups were comparable in terms of mean lactate levels. However, in both groups, the peak rise of lactate levels was seen in the immediate postoperative period. Out of 20 patients in Group 1, nine patients received bicarbonate, and out of 20 patients in Group 2, only three received bicarbonate, and the difference was statistically significant (p < 0.05). Table 5 depicts the postoperative liver function tests at various intervals. Both groups showed an increasing trend in PT. The highest mean PT was seen on day 3, followed by a declining trend. The highest mean bilirubin was seen on day 1 in both groups. There was a peak rise in SGOT/PT in the immediate postoperative period in both groups, followed by a decreasing trend and normalization on day 5 of surgery.

**Table 1:** Characteristics of the study population Values are presented as mean ± SD. A p-value of less than 0.05 is considered significant.

Variables		Group 1 (n = 20)	Group 2 (n = 20)	p-value
Gender	Female	10 (50%)	11 (55%)	0.285
	Male	10 (50%)	9 (45%)	
Age (in years), mean ± SD		36.55 ± 9.838	32.85 ± 11.681	0.285
Weight (in kg), mean ± SD		66.20 ± 9.328	63.35 ± 9.005	0.331

**Table 2:** Hemodynamic parameters and urine output of the study population measured at various intervals Values are presented as mean ± SD. A p-value of less than 0.05 is considered significant.

Groups	T0	T1	T2	T3	T4	T5
Heart rate (beats per minute) (mean ± SD)						
Group 1	75.05 ± 12.094	84.70 ± 9.021	92.35 ± 10.820	90.15 ± 12.162	86.40 ± 15.017	77.05 ± 10.904
Group 2	73.05 ± 11.980	88.10 ± 13.467	94.65 ± 14.394	89.45 ± 9.434	89.10 ± 13.954	83.30 ± 11.886
p-value	0.602	0.354	0.571	0.84	0.559	0.091
Mean arterial pressure (mm Hg) (mean ± SD)						
Group 1	78.70 ± 13.436	86.15 ± 12.08	84.30 ± 9.921	86.80 ± 13.942	86.65 ± 12.97	88.10 ± 13.58
Group 2	79.44 ± 14.337	81.94 ± 10.788	80.56 ± 11.81	79.06 ± 18.095	89.28 ± 15.30	83.83 ± 13.997
p-value	0.87	0.253	0.295	0.07	0.57	0.347
Hemoglobin (gm%) (mean ± SD)						
Group 1	12.745 ± 2.0498	13.005 ± 1.837	11.98 ± 2.01	10.995 ± 1.74	10.720 ± 1.711	10.64 ± 1.663
Group 2	12.345 ± 1.6785	12.610 ± 1.742	11.54 ± 1.88	11.220 ± 1.80	10.555 ± 1.67	10.30 ± 1.474
p-value	0.242	0.204	0.182	0.147	0.429	0.664
Hematocrit (mean ± SD)						
Group 1	38.389 ± 6.135	38.842 ± 5.607	35.989 ± 6.19	32.989 ± 5.38	32.053 ± 5.25	31.932 ± 5.12
Group 2	37.029 ± 4.90	38.057 ± 5.201	34.567 ± 5.44	33.729 ± 5.33	31.786 ± 4.91	30.929 ± 4.31
p-value	0.441	0.649	0.444	0.666	0.869	0.506
Urine output (ml) (mean ± SD)						
Group 1	95.75 ± 76.26	261 ± 217.3	210 ± 170.21	315 ± 164.717	460 ± 219.80	1305 ± 388.95
Group 2	74.25 ± 64.97	198.25 ± 143.74	313 ± 170.04	351.25 ± 235.70	510.25 ± 290.04	1302.5 ± 377.83
p-value	0.343	0.288	0.063	0.576	0.541	0.984

**Table 3:** Acid-base parameters of the study population measured at various intervals Values are presented as mean ± SD. A p-value of less than 0.05 is considered significant. * Significant p-values

Groups	T0	T1	T2	T3	T4	T5
pH (mean ± SD)						
Group 1	7.44370 ± 0.037	7.40185 ± 0.058	7.3876 ± 0.039*	7.3737 ± 0.030*	7.3694 ± 0.029*	7.381 ± 0.02*
Group 2	7.42615 ± 0.050	7.40860 ± 0.036	7.3943 ± 0.046	7.39270 ± 0.510	7.38760 ± 0.051	7.4245 ± 0.03
p-value	0.221	0.664	0.629	0.16	0.177	0
PaCO_2_ (mm Hg) (mean ± SD)						
Group 1	33.185 ± 4.104	35.215 ± 6.476	34.325 ± 3.239	35.665 ± 3.242	35.540 ± 2.413	35.855 ± 3.86
Group 2	34.810 ± 3.693	33.735 ± 3.157	35.580 ± 3.426	35.670 ± 5.055	36.205 ± 4.516	36.075 ± 4.23
p-value	0.196	0.364	0.241	0.997	0.565	0.865
HCO₃ (mmol/L) (mean ± SD)						
Group 1	21.43 ± 1.538	20.365 ± 1.622	19.315 ± 1.635	19.305 ± 1.508	19.625 ± 1.498	21.585 ± 1.82
Group 2	21.38 ± 1.594	20.4 ± 1.594	20.58 ± 2.025	21.465 ± 2.038	21.805 ± 1.404	23.104 ± 2.22
p-value	0.92	0.945	0.036*	0.000*	0.000*	0.024*
Anion gap (mean ± SD)						
Group 1	10.38 ± 3.080	11.46 ± 2.451	10.23 ± 2.719	10 ± 2.45	9.495 ± 2.170	9.58 ± 2.09
Group 2	10.18 ± 1.501	11.71 ± 3.084	11.32 ± 3.49	11.69 ± 4.007	10.9 ± 1.849	9.68 ± 1.511
p-value	0.796	0.778	0.275	0.116	0.034*	0.863
Base excess (mean ± SD)						
Group 1	-2.27 ± 1.68	-4.240 ± 0.097	-5.360 ± 1.73	-5.110 ± 1.43	-4.590 ± 1.97	-2.735 ± 1.497
Group 2	-2.75 ± 1.34	-4.005 ± 1.91	-3.630 ± 1.88	-3.205 ± 1.87	-3.350 ± 1.73	-1.565 ± 1.704
p-value	0.326	0.628	0.005*	0.001*	0.041*	0.027*

**Table 4:** Serum electrolytes of the study population measured at various intervals Values are presented as mean ± SD. A p-value of less than 0.05 is considered significant. * Significant p-value

Groups	T0	T1	T2	T3	T4	T5
Sodium (mEq/L) (mean ± SD)						
Group 1	136.69 ± 1.984	137.89 ± 2.60	138.330 ± 1.84	138.43 ± 1.73	137.485 ± 1.75	136.73 ± 1.787
Group 2	135.39 ± 2.553	136.94 ± 2.64	136.015 ± 2.87	135.945 ± 2.75	135.97 ± 1.81	135.82 ± 1.885
p-value	0.08	0.259	0.004*	0.002*	0.011*	0.125
Chlorine (mEq/L) (mean ± SD)						
Group 1	107.15 ± 1.981	110.2 ± 3.02	113.8 ± 3.318	113.9 ± 2.404	113.55 ± 2.85	110.9 ± 3.40
Group 2	106.60 ± 2.62	105.15 ± 2.05	104.75 ± 2.314	104.4 ± 2.257	105.35 ± 1.81	103.65 ± 2.455
p-value	0.062	0.000*	0.000*	0.000*	0.000*	0.000*
Lactate (mEq/L) (mean ± SD)						
Group 1	1.753 ± 0.694	1.9955 ± 0.68	2.417 ± 0.808	3.142 ± 0.774	3.300 ± 0.468	2.281 ± 0.732
Group 2	1.7875 ± 0.591	2.1235 ± 0.85	2.7295 ± 1.29	3.145 ± 1.157	3.471 ± 0.917	2.063 ± 0.592
p-value	0.867	0.603	0.366	0.994	0.462	0.307

**Table 5:** Postoperative liver function tests of the study population measured at various intervals Values are presented as mean ± SD. A p-value of less than 0.05 is considered significant.

Groups	Day 0	Day 1	Day 2	Day 3	Day 4	Day 5
Bilirubin (mg/dL) (mean ± SD)						
Group 1	2.1855 ± 0.903	2.8295 ± 1.12	2.597 ± 0.91	2.134 ± 0.71	1.549 ± 0.668	1.044±0.626
Group 2	2.1995 ± 0.82	2.6915 ± 1.11	2.381 ± 1.08	2.041 ± 1.26	1.514 ± 1.06	1.09 ± 0.179
p-value	0.96	0.699	0.499	0.776	0.902	0.841
SGOT (IU/L) (mean ± SD)						
Group 1	302.75 ± 135	286.9 ± 144.3	185.85 ± 106	117.55 ± 70	76.8 ± 48.122	60.6 ± 36.01
Group 2	279.70 ± 165	239.5 ± 115.4	159.3±77.08	101.39 ± 49	84.35 ± 34.63	66.7 ± 29.90
p-value	0.632	0.259	0.391	0.404	0.572	0.564
PT						
Group 1	15.57 ± 4.42	17.335 ± 3.59	18.07 ± 3.48	17.14 ± 3.89	14.745 ± 2.82	13.54 ± 2.40
Group 2	15.79 ± 3.15	20.5 ± 3.17	19.51 ± 4.49	15.79 ± 3.20	13.37 ± 1.73	12.48 ± 1.19
p-value	0.857	0.005	0.265	0.239	0.071	0.085

## Discussion

The aim of the study was to assess the effect of a 0.9% normal saline versus an acetate-gluconate-based balanced solution on acid-base status and postoperative liver function in 40 patients undergoing donor hepatectomy. It was observed that both 0.9% normal saline and PlasmaLyte maintained hemodynamic stability. We aimed for a mean arterial pressure (MAP) of 60 mm Hg to avoid hypoperfusion of the liver and a possible increase in lactate levels following hypoperfusion. None of the patients in either group required the use of vasopressors to maintain MAP within the normal range. Excessive volume loading was avoided to decrease venous bleeding and graft congestion. Surgical interventions like the application of temporary vascular occlusion and modifications like a harmonic scalpel and Cavitator ultrasonic aspirator were used along the plane of resection.

We found that levels of lactate during the intraoperative period or in the postoperative period were not significantly different between the two groups. However, as expected in any major hepatectomy, there was a sequential rise in serum lactate levels from baseline through the course of surgery, with a peak at the end of surgery in both groups. Perioperative lactic acidosis is seen to add to donor morbidity. The levels of lactate have been used to predict recovery in hepatic surgeries and other abdominal surgeries [11-13]. Lactate is converted to pyruvate and generates adenosine triphosphate (ATP) by oxidative phosphorylation in the mitochondria. A portion of the lactate is also metabolized by the Cori cycle to generate glucose with the consumption of ATP [14]. Although the liver has a large functional reserve and can metabolize endogenous lactate after major hepatectomy, lactate levels can rise if the volume of the liver is inadequate, as in situations following major hepatectomy [15] and have been shown to have a close association with sequential organ failure assessment scores in intensive care patients [16]. Although lactate levels were not different in the two groups, we found significant differences in bicarbonate ion, anion gap, and base excess. Patients who received normal saline during the study had a significant fall in bicarbonate and base excess during the course of surgery, while patients who received PlasmaLyte during the surgery showed an improved profile with regard to bicarbonate, anion gap, and base excess. This could have been due to the administration of a balanced salt solution (PlasmaLyte) containing metabolizable anions, acetate, and gluconate in Group 2, which acts as a buffer and converts into bicarbonate in the body. An observational study from a database of patients undergoing abdominal gynecologic surgery showed a similar profile and concluded that acetate-based solutions were better in terms of stability of pH and HCO₃ [10].

We had 12 patients out of 40 in whom base excess was less than -6, of which nine were from the group receiving normal saline. Volta et al. [17] in their double-blinded randomized trial of 40 patients undergoing major abdominal surgery concluded that a strategy of fluid replacement based on normal saline was associated with a lower pH and a lower concentration of bicarbonate and base excess. This compared well with our study. We did not find any difference in terms of pH in the two groups intraoperatively except at the end of 24 hours, where patients who received PlasmaLyte showed pH values higher (mean pH 7.48 in comparison to 7.38 in normal saline; p < 0.05). The normalization of pH in normal saline at the end of 24 hours could have been due to the supplementation of bicarbonate in this group of patients to correct acidosis and redistribution of body fluids and recovery from acidosis. A total of nine patients required supplementation of 7.5% w/v sodium bicarbonate in the normal saline group, while only three patients in the PlasmaLyte group required supplementation of sodium bicarbonate (p < 0.05). We found higher sodium in the group receiving normal saline, which was statistically significant, and this higher trend was seen in the intraoperative period. This finding compares well with a study by Lehmann et al. [18], where it was found that a saline-based infusion regimen in patients with subarachnoid hemorrhage was associated with a higher sodium input (although normal average sodium) compared to sodium excretion during the first 24 hours. The study was limited by a small sample size. We found higher chloride levels in the group receiving normal saline as compared to the PlasmaLyte group. This increasing trend of chloride level continued even in the postoperative period. This was probably due to the higher chloride content of normal saline. Hyperchloremic acidosis was seen in patients who received normal saline, and this goes well with Orbegozo Cortés et al. [19], where 22 of 23 articles explored the effects on acid-base status and showed that the administration of normal saline was associated with an increase in chloride levels.

In our study in all patients, liver functions were followed for five days starting on the day of surgery. Postoperative liver functions in both groups were comparable, and no statistically significant difference was found in the two groups. This is comparable to a study by Kumar et al. [20], where no difference was found in the two groups with regard to postoperative liver functions. Shin et al. [21] reported that the use of Ringer’s lactate may have been associated with hepatic dysfunction, as measured by a transient increase in bilirubin and mild prolongation of prothrombin time during the first two days. We found similar trends in postoperative liver functions in both groups. By summarizing the above observations, it can be concluded that changes in acid balance noted during the study may be due to administered solutions. PlasmaLyte or acetate-containing solutions were able to maintain a better metabolic profile and acid-base status of the patient compared to 0.9% normal saline, and we did not find any major difference in postoperative liver function.

Limitations

This study included a small patient cohort, with participants allocated alternately to each group; however, randomization could have been a better method of allocation. Intraoperative management of donors was determined by individual anesthesiologists, and the fluid administered during the procedure was open-label. However, the data were objectively collected through arterial blood sample analysis, minimizing the potential for observer bias.

## Conclusions

In this study, we compared the effects of 0.9% normal saline and acetate-gluconate-based balanced solution (PlasmaLyte A®) on acid-base status and liver function in patients undergoing living donor hepatectomy. Our findings demonstrated that the use of an acetate-gluconate-based balanced solution resulted in better preservation of acid-base balance in terms of base excess, anion gap, and bicarbonate and a lesser need to correct the metabolic acidosis by supplementation of sodium bicarbonate. Both solutions were comparable in terms of postoperative liver function. These results suggest that acetate-gluconate-based balanced solutions may offer superior intraoperative and postoperative management, leading to safer and more effective fluid therapy in liver transplantation surgery. Further large-scale studies are warranted to confirm these findings and potentially update clinical practice guidelines.
